# Intestinal Immune System Expression of Coho Salmon Challenged with Oxytetracycline: *In Vivo* and *In Vitro* Approach

**DOI:** 10.3390/ijms26136330

**Published:** 2025-06-30

**Authors:** Daniela Nualart, José Luis P. Muñoz, Luis Vargas-Chacoff

**Affiliations:** 1 Instituto de Ciencias Marinas y Limnológicas, Laboratorio de Fisiología de Peces, Universidad Austral de Chile, Valdivia 5090000, Chile; luis.vargas@uach.cl; 2 Millennium Institute Biodiversity of Antarctic and Subantarctic Ecosystems, BASE, University Austral of Chile, Valdivia 5090000, Chile; 3 Centro FONDAP de Investigación en Dinámica de Ecosistemas Marinos de Altas Latitudes (IDEAL), Universidad Austral de Chile, Valdivia 5090000, Chile; 4 Centro de Investigación y Desarrollo I~Mar, Universidad de Los Lagos, Puerto Montt 5480000, Chile; 5 Integrative Biology Group, Universidad Austral de Chile, Valdivia 5090000, Chile

**Keywords:** antibiotics, cell culture, stress, aquaculture, salmon

## Abstract

Oxytetracycline (OTC) has served as an antibiotic to treat various bacterial infections in fish raised in aquaculture. Nonetheless, administering OTC in overly high doses can lead to adverse side effects in fish and also negatively impact on their surroundings. The objective of this work was to evaluate the expression levels of immune markers such as TLR-1, TLR-2, IκB-α, MyD88, NF-κB, IFN-γ, and IL-6 in intestinal cell primary culture (foregut, midgut, and hindgut) using qRT-PCR, and in addition, to assess the *in vivo* response to different doses of OTC in coho salmon at different times. The expression levels of all genes increased significantly after 1 h on day 1 with high doses of OTC compared with control conditions in all tissues under both approaches (*in vivo* and *in vitro*). However, the transcriptional responses decreased to 3, 6, and 12 h *in vitro* and day 3 *in vivo*. In conclusion, the transcriptional responses were differentially modulated by OTC in the three intestinal portions under both experimental conditions. These results demonstrate for the first time in primary cell culture fish that the expression of immune biomarkers in all tissues induces a differential response of these genes, depending on the concentration of OTC and the kinetics of time. This study offers valuable insights that can be applied to enhance aquaculture, determine optimal drug doses, and improve fish health.

## 1. Introduction

The immune system is divided into two primary sections: the innate immune system and the adaptive immune system. The innate immune system serves as the first line of defense against infections and illnesses, but it lacks the ability to remember previous encounters [1,2]. On the other hand, the adaptive immune system is designed to target specific antigens and can provide lasting immunity [3]. This aspect of the immune system is vital for the well-being and survival of fish in aquatic environments. The immune response in fish is a sophisticated process that involves the coordination of various tissues and cells, such as those found in the head kidney and spleen. The head kidney has a hematopoietic function; it is the principal organ for phagocytosis and antigen processing and is the primary site for antibody production [4,5]. The spleen also functions as a major secondary immune organ and plays a significant role in the clearance of bloodborne antigens and antigen presentation and initiation of adaptive immune response [6]. Both tissues play a crucial role in defending against pathogens and regulating the immune response [7,8,9]. However, several external issues, including environmental factors, biological factors, stress, and internal factors such as genetic makeup, age, sex, and maternal effects, can affect the immunological defense capabilities of fish [7,10,11]. Temperature, salinity, and stress can significantly affect the immune response of fish [12,13,14]. For example, changes in temperature and oxygen levels can alter the expression of immunity-related genes [15,16,17], while salinity can affect the function of immune cells in *Scatophagus argus* [18]. Furthermore, chronic stress can weaken the fish’s immune system, making them more susceptible to disease [19,20]. Stress is an occurrence that many animals undergo, triggering a variety of reactions that engage the three main regulatory systems: the neural, endocrine, and immune systems. In situations where the stress factor is acute and of short duration, the response pattern tends to be stimulating, and the immune response in fish enters an activating phase that primarily boosts innate responses [20]. In this sense, stress may cause high consumption of energy reserves with reallocation of metabolic energy, which interferes negatively with physiological processes such as immunological capacity, influencing the ability to resist infection processes by modulating several immune system components [11]. If the stressor is chronic, the immune response exhibits suppressive effects, and consequently, the likelihood of infection may be increased [21]. For this reason, the use of antibiotics in the diet of salmon, particularly oxytetracycline [22], for both therapeutic and prophylactic purposes have been shown to pose an increasing challenge in the aquaculture industry [23]. Oxytetracycline (OTC) is one of the most used antibiotics in aquaculture [24]. Owing to its broad antibiotic spectrum and low cost [25], it is widely used to prevent and treat bacterial fish diseases [26]. However, this use can have a negative impact on fish health and the environment. Prolonged use can lead to antibiotic resistance, posing a significant threat to global health [27]. Although interactions between antibiotics and the fish immune system have been reported in the literature in turbot, sea bream, Atlantic cod, rainbow trout, gilthead seabream, and coho salmon [28,29,30,31,32], the potential negative effects of these therapeutics on fish immune responses and health have been largely overlooked. In particular, eliminating pathogenic bacteria using antibiotic treatments requires a well-functioning fish immune system [33]. The presence of immune cells and their response have been well studied in the gut of common carp [34], European sea bass [35,36], and Atlantic salmon [37]. In addition, antibiotics may negatively affect gut bacterial diversity in *Salmo salar* [38,39] and the liver and antioxidant systems [40], which can indirectly impact the fish’s immune function. The intestine is a crucial component of the fish digestive system, actively promoting the fish’s healthy growth and development and serving as a vital immune barrier [34,35,36,37,38,39,40,41]. Moreover, previous functional studies based on transcriptomic analyses have attributed distinct functions to the segments of the European seabass intestine [41,42]. The different sections of the fish’s gut (anterior, middle, and posterior) play a critical role in nutrient absorption and regulating the immune response. Each intestinal region fulfills specific physiological functions, including digestion, absorption, regulation, and balance, and contributes to the immune system. In addition, gene expressions have been shown to vary between these portions depending on the type of stressor or pathogen present [43].

Exposure to environmental factors and the use of antibiotics can impact the function and structure of these intestinal portions, potentially leading to negative consequences for the fish’s health. This work aimed to evaluate the expression levels of immune markers such as TLR-1, TLR-2, IκB-α, MyD88, NF-κB, IFN-γ, and IL-6 in the foregut, midgut, and hindgut of coho salmon exposed to different doses of OTC under two approaches (*in vitro* and *in vivo*).

## 2. Results

### 2.1. In Vitro Experiments

#### 2.1.1. Foregut

**Figure 1:**
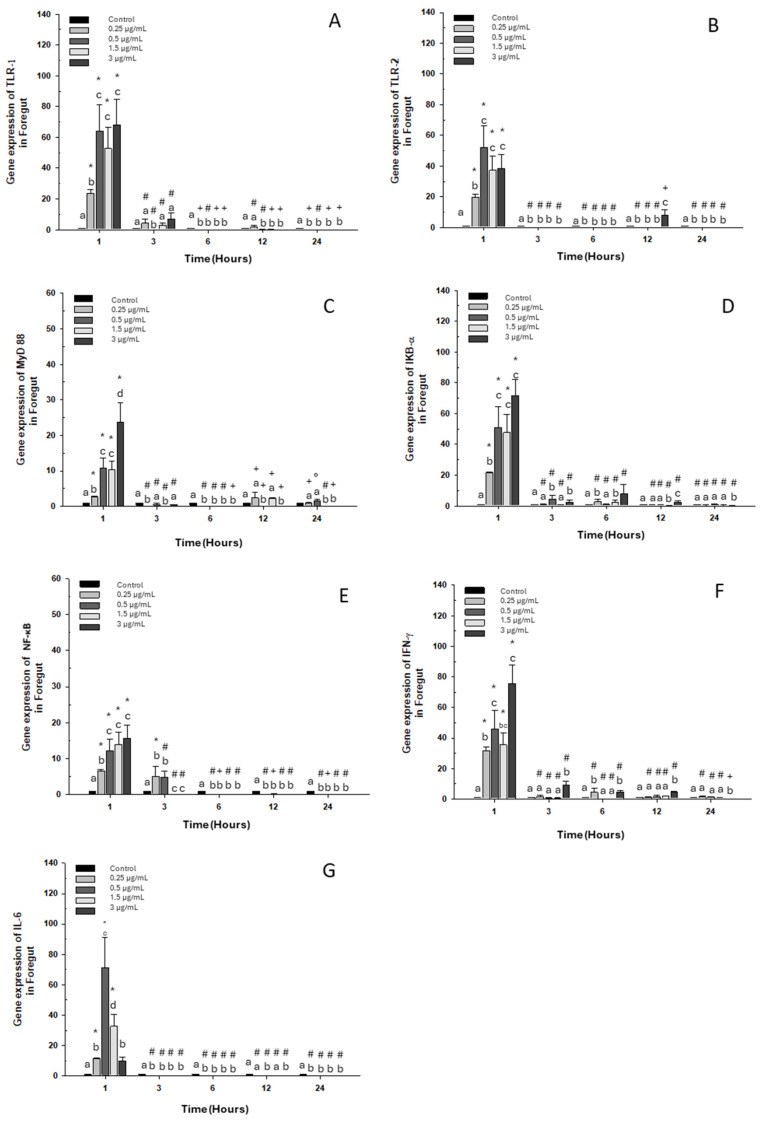
Gene expression in the primary culture of the foregut of *O. kisutch* treated with oxytetracycline (OTC). (**A**) TLR-1, (**B**) TLR-2, (**C**) MyD88, (**D**) IκB-α, (**E**) NF-κb, (**F**) INF-γ, and (**G**) IL-6. The relative expression of genes was calculated using the comparative Ct method (2^−ΔΔCT^), with the 18S ribosomal protein as the internal reference gene. Each value represents the mean ± S.E.M. Different letters (a,b,c) indicate statistical differences at the same time point and between treatments. Symbols (*, #, +) over the bars indicate statistical differences of treatments (control and different doses of antibiotic) at different time points. Two-way ANOVA followed by Tukey’s test (*p* < 0.05).

When evaluating the gene expression of different immune markers, we observed a similar pattern in each gene assayed, with a significant increase at the first hour with respect to the control group for all doses of OTC (Figure 1).

The expression of toll-like receptor 1 “TLR-1” also increased at 3 h at the highest dose (Figure 1A). As for TLR-2, its expression increased only at 12 h at the highest dose (Figure 1B). Expressions of myeloid differentiation primary response 88 “MyD 88” also increased at 12 h, at the lowest dose 0.25 μg/mL and at 1.5 μg/mL (Figure 1C). Likewise, an increase in the transcripts of inhibitor α or “IκB-α” was observed at 3, 6 and 12 h with doses of 0.5, 0.25, and 3 μg/mL, respectively (Figure 1D). On the other hand, nuclear factor κB “NF-κB” maintained its high expression at 3 h with the lowest doses of 0.25 μg/mL and 0.5 μg/mL (Figure 1E). Interferon-gamma mRNA “INF-γ” increased in expression at 3, 6, and 12 h of exposure to the high dose (Figure 1F). Finally, the expression of interleukin 6 (IL-6) decreased significantly at 3, 6, 12, and 24 h of exposure to all doses (Figure 1G).

#### 2.1.2. Midgut

**Figure 2:**
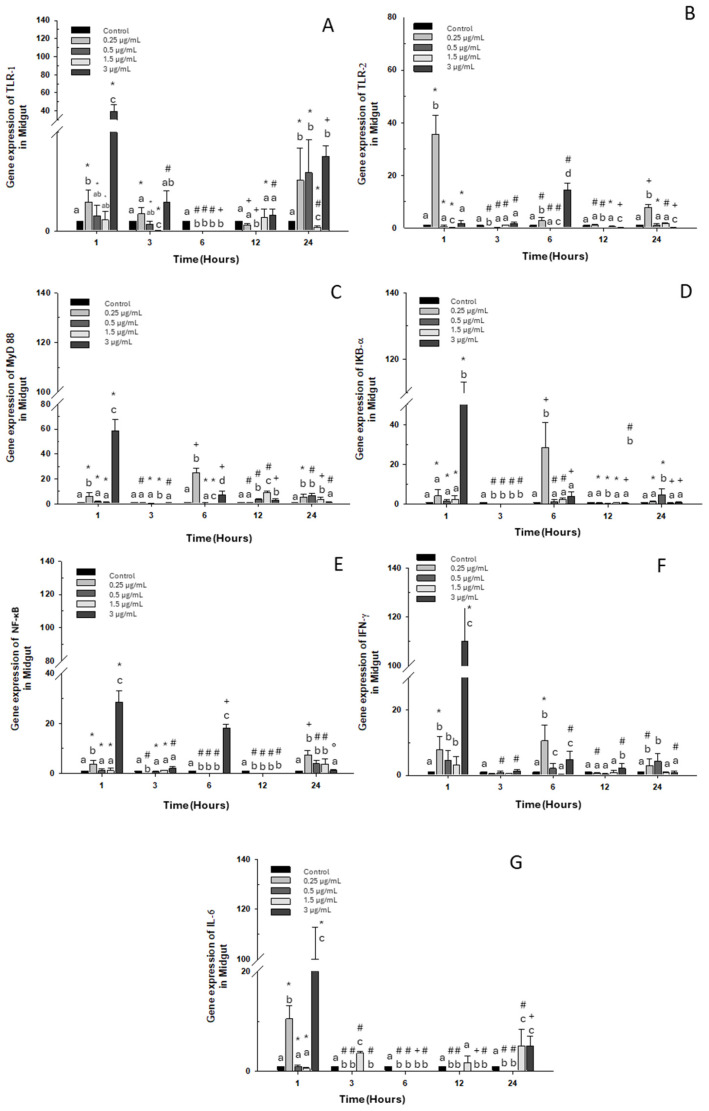
Gene expression in the primary culture of the midgut of *O. kisutch* treated with oxytetracycline (OTC). (**A**) TLR-1, (**B**) TLR-2, (**C**) MyD88, (**D**) IκB-α, (**E**) NF-κb, (**F**) INF-γ, and (**G**) IL-6. The relative expression of genes was calculated using the comparative Ct method (2^−ΔΔCT^), with the 18S ribosomal protein as the internal reference gene. Each value represents the mean ± S.E.M. Different letters (a,b,c) indicate statistical differences at the same time point and between treatments. Symbols (*, #, +) over the bars indicate statistical differences of treatments (control and different doses of antibiotic) at different time points. Two-way ANOVA followed by Tukey’s test (*p* < 0.05).

A significant increase in TLR-1 transcripts at 1 and 24 h was observed compared to the control group with the high dose of OTC (3 μg/mL), as well as with doses of 0.25 and 0.5 μg/mL (Figure 2A). As for TLR-2 expression, it only increased with the 0.25 μg/mL dose at 1, 3, and 24 h. However, at 6 h, its expression only increased when exposed to the highest dose of 3 μg/mL of OTC (Figure 2B). MyD 88 and IKB-α expressions increased with exposure to the highest dose of 3 μg/mL at 1 h and with the lowest dose of 0.25 μg/mL at 6 h. (Figure 2C,D). Similarly, at 1 and 6 h of exposure to OTC, the mRNA of NF-κB increased; however, at 12 h, its expression decreased significantly. The other experimental doses (low and intermediate) caused increases only at 24 h (Figure 2E). On the other hand, INF-γ mRNA increased at 1 and 6 h with the highest OTC dose of 3 μg/mL and at 6 and 24 h of exposure to the low dose of 0.25 μg/mL (Figure 2F). IL-6 expression also increased significantly at 3, 12, and 24 h of exposure to the intermediate dose of 1.5 μg/mL. The high dose increased the transcripts of this gene at 1 and 24 h of exposure (Figure 2G).

#### 2.1.3. Hindgut

**Figure 3:**
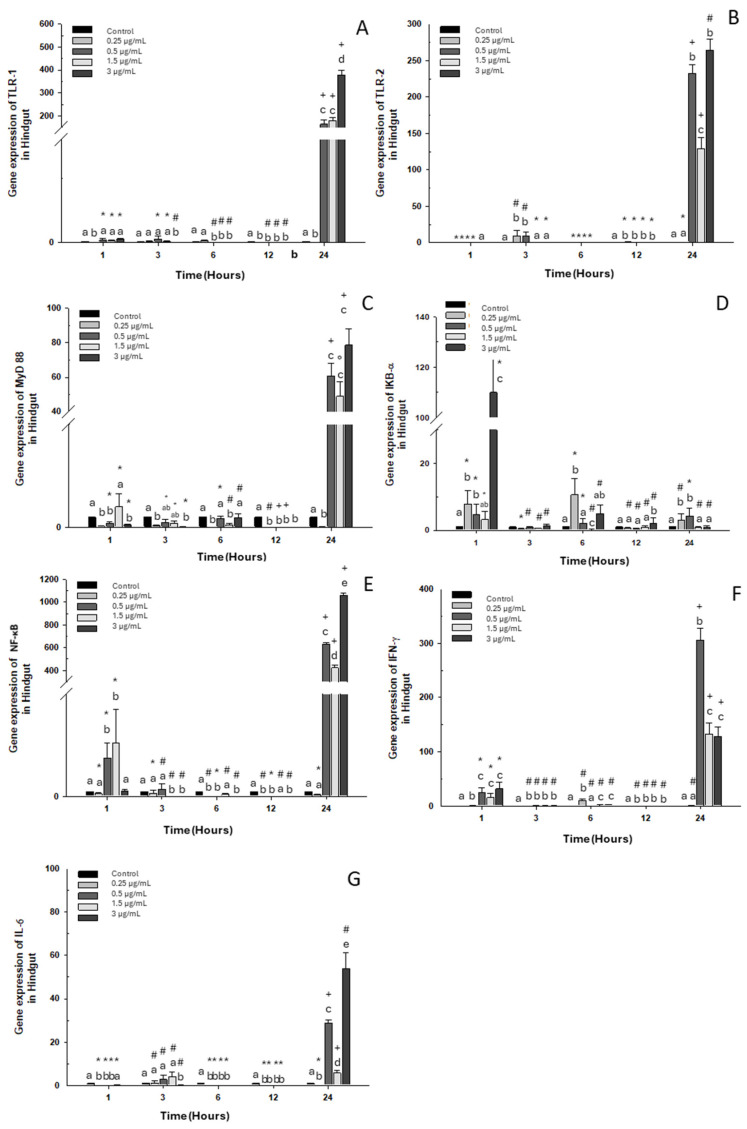
Gene expression in the primary culture of the hindgut of *O. kisutch* treated with oxytetracycline (OTC). (**A**) TLR-1, (**B**) TLR-2, (**C**) MyD88, (**D**) IκB-α, (**E**) NF-κb, (**F**) INF-γ, and (**G**) IL-6. The relative expression of genes was calculated using the comparative Ct method (2^−ΔΔCT^), with the 18S ribosomal protein as the internal reference gene. Each value represents the mean ± S.E.M. Different letters (a–e) indicate statistical differences at the same time point and between treatments. Symbols (*, #, +) over the bars indicate statistical differences of treatments (control and different doses of antibiotic) at different time points. Two-way ANOVA followed by Tukey’s test (*p* < 0.05).

A similar pattern was noted in all markers, where overexpression was seen at 24 h of exposure to OTC at the three highest doses in the experiment (0.5, 1.5, and 3 μg/mL) compared to the control group (Figure 3), with just one exception: IKB-α. Downregulation was observed in TLR-1 expression at 1, 3, 6, and 12 h of exposure to all concentrations of OTC used in the *in vitro* experiment (Figure 3A). TLR-2 and IL-6 expression increased only at 3 h of exposure to doses of 0.25, 0.5, and 1.5 μg/mL of OTC (Figure 3B,G). On the other hand, MyD88 and NF-κB expression decreased at 1, 3, 6, 12, and 24 h with a dose of 0.25 μg/mL of OTC (Figure 3C,E). The highest level of IKB-α expression occurred after one hour of exposure (Figure 3D). IFN-γ also showed overexpression at 1 h with the highest doses (Figure 3E). Finally, IL-6 expression showed downregulation throughout the kinetics of *in vitro* exposure to OTC, except at 24 h, where a significant increase was observed with doses of 0.5, 1.5, and 3 μg/mL of OTC.

### 2.2. In Vivo Experiment

#### 2.2.1. Foregut

**Figure 4:**
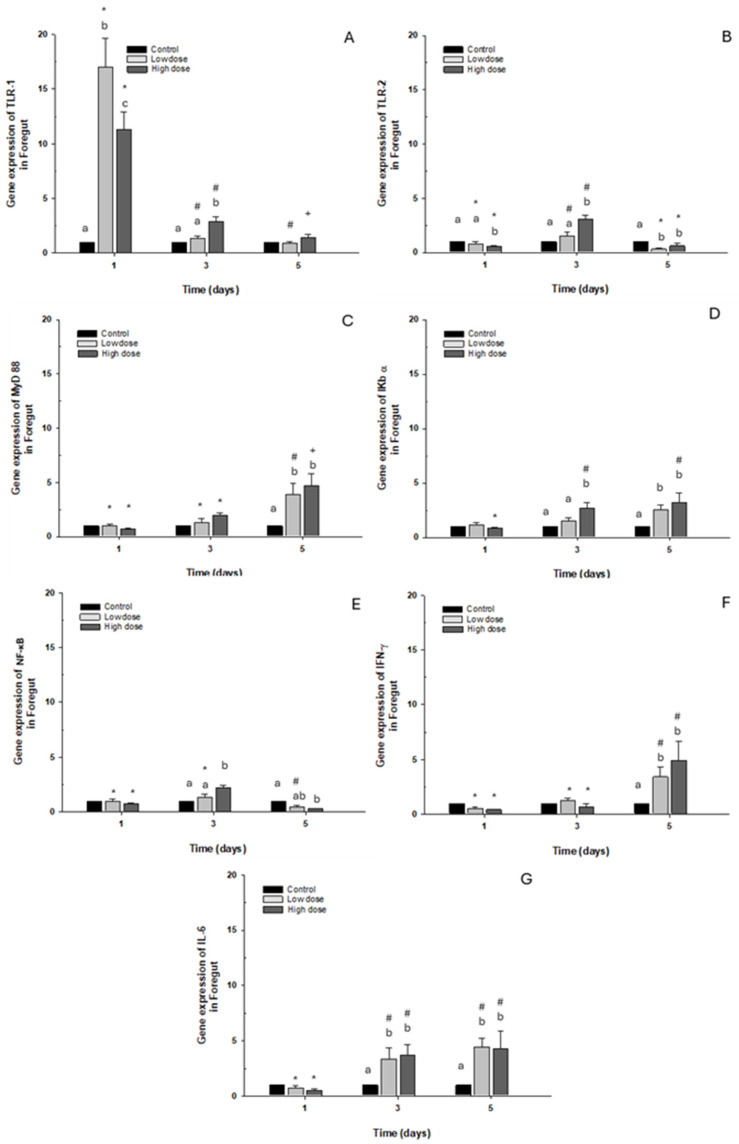
Gene expression *in vivo* of the foregut of *O. kisutch* treated with oxytetracycline (OTC). (**A**) TLR-1, (**B**) TLR-2, (**C**) MyD88, (**D**) IκB-α, (**E**) NF-κb, (**F**) INF-γ, and (**G**) IL-6. The relative expression of genes was calculated using the comparative Ct method (2^−ΔΔCT^), with the 18S ribosomal protein as the internal reference gene. Each value represents the mean ± S.E.M. Different letters (a,b,c) indicate statistical differences at the same time point and between treatments. Symbols (*, #, +) over the bars indicate statistical differences of treatments (control and different doses of antibiotic) at different time points. Two-way ANOVA followed by Tukey’s test (*p* < 0.05).

The toll-like receptor TLR-1 presented overexpression on day 1 in both cases in the control group after both doses, with the gene expression levels decreasing (Figure 4A). Regarding expression of TLR-2 and NF-κB, only a significant 2.5-fold increase was observed compared to the control group when exposed to the high dose. However, on day 5, a significant decrease was observed with both OTC doses (Figure 4B,E). When transcript levels of myeloid differentiation primary response 88 (MyD88) and interferon-gamma (IFN-γ) were analyzed, they increased significantly, about 5-fold on day 5 with low and high doses of OTC, compared with the control group (Figure 4C,F). Concerning the levels of NF-κB inhibitor α (IκB-α) and interleukin 6 (IL-6), there was an elevation observed at 3 and 5 days following stimulation with both low and high doses, showing less than a fivefold increase compared to the control condition (Figure 4D,G).

#### 2.2.2. Midgut

**Figure 5:**
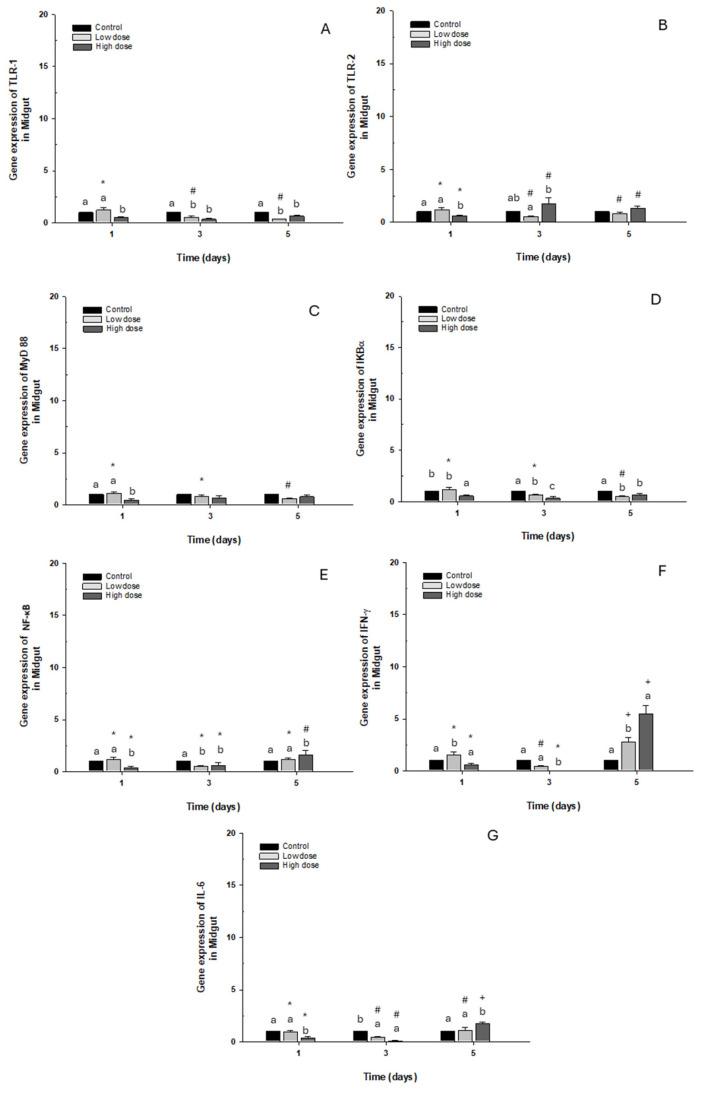
Gene expression *in vivo* of the midgut of *O. kisutch* treated with oxytetracycline (OTC). (**A**) TLR-1, (**B**) TLR-2, (**C**) MyD88, (**D**) IκB-α, (**E**) NF-κb, (**F**) INF-γ, and (**G**) IL-6. The relative expression of genes was calculated using the comparative Ct method (2^−ΔΔCT^), with the 18S ribosomal protein as the internal reference gene. Each value represents the mean ± S.E.M. Different letters (a,b,c) indicate statistical differences at the same time point and between treatments. Symbols (*, #, +) over the bars indicate statistical differences of treatments (control and different doses of antibiotic) at different time points. Two-way ANOVA followed by Tukey’s test (*p* < 0.05).

TLR-1, TLR-2, MyD88, IKB-α, NFK-β, and IL-6 gene expression presented a similar pattern to the control group. At the same time, IFN-γ on day 5 showed a significant “3- to 5-fold” increase in fish exposed to low and high doses compared to the control group (Figure 5A–F).

#### 2.2.3. Hindgut

**Figure 6:**
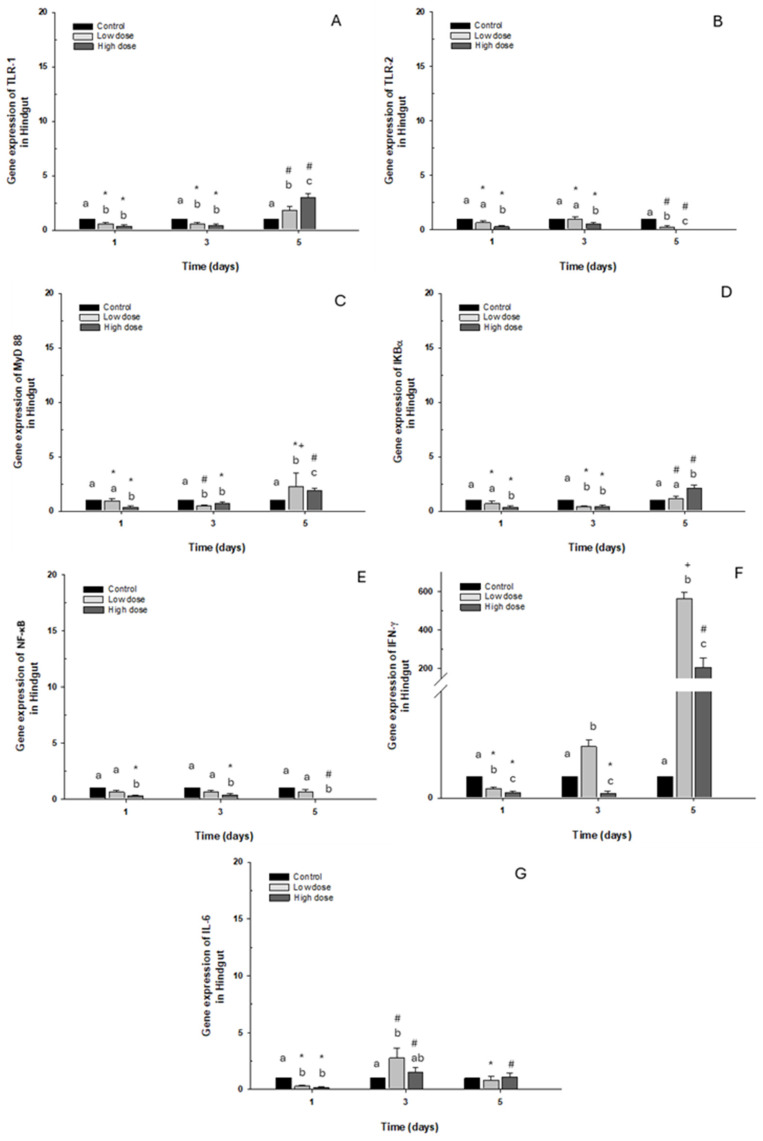
Gene expression *in vivo* of the hindgut of *O. kisutch* treated with oxytetracycline (OTC). (**A**) TLR-1, (**B**) TLR-2, (**C**) MyD88, (**D**) IκB-α, (**E**) NF-κb, (**F**) INF-γ, and (**G**) IL-6. The relative expression of genes was calculated using the comparative Ct method (2^−ΔΔCT^), with the 18S ribosomal protein as the internal reference gene. Each value represents the mean ± S.E.M. Different letters (a,b,c) indicate statistical differences at the same time point and between treatments. Symbols (*, #, +) over the bars indicate statistical differences of treatments (control and different doses of antibiotic) at different time points. Two-way ANOVA followed by Tukey’s test (*p* < 0.05).

Gene expression of TLR-1 and IκB-α in this intestinal portion decreased significantly with both doses on days 1 and 3. On the contrary, an increase was observed on day 5 in fish (approx. 3-fold) exposed to the high dose compared to the control group (Figure 6A,D). Regarding TLR-2 and NF-κB expression, downregulation was observed compared to the control group when the fish were exposed to the high dose, but only NF-κB presented statistical differences (Figure 6B,E). Additionally, MyD88 expression increased only on day 5 following exposure to the high dose of OTC. However, a decrease was observed on day 1 and day 3 when the fish were exposed to the high and low doses, respectively (Figure 6C). The immune marker with the highest expression was IFN-γ, where overexpression was significant on day 3 and day 5 post-exposure, increasing more than 100- and 400-fold, compared to the control group (Figure 6F). Finally, IL-6 expression decreased significantly on day 1 of exposure to both doses. Contrary to day 3, it was increased at low doses (Figure 6G).

## 3. Discussion

Research on the immune response in fish, particularly in salmonid species, has proven essential for understanding how antibiotic treatments, such as OTC, affect health and immunity. In this study, we present new findings on the immune response of coho salmon, explicitly examining the expression of immune genes of the intestine in three intestinal portions following exposure to varying doses of OTC using two approaches: *in vivo* and *in vitro*.

The intestine has the highest number of immune cells in any body tissue. It is constantly exposed to a variety of antigens and potential immune triggers. However, many studies overlook that the intestinal tract comprises different regions with distinct anatomical and physiological characteristics and gene expression patterns [44]. In particular, we highlight that the transcriptional responses of genes such as IL-6, IFN-γ, and NF-κB can present peaks of activation followed by upregulation, which is consistent with the literature on immunotoxicology in fish [26,28]. The observed variability may also be influenced by intestinal segmentation (foregut, midgut, hindgut), since each region has a different immune cell density, associated microbiota, and absorption capacity, factors that affect gene expression kinetics [39,41].

The *in vitro* experiment revealed a pattern of increase at early activation; at 1 h of exposure to OTC, the highest doses, ranging from 0.5 to 3 μg/mL, showed the most significant effect in the anterior intestinal part (foregut), also exhibiting a dose-dependent pattern in all immune markers. This finding is consistent with the study by Tafalla et al. (1999) [28], in which OTC was administered orally over 12 days and combined with OTC baths on days 1, 3, and 5. Observations showed that after exposure, the head kidney macrophages’ respiratory burst and phagocytosis were restricted by the *in vitro* treatment, and this was dose-dependent for several immune functions in turbot (*Scophthalmus maximus* L.). Several studies have shown the activation of innate immunity in fish exposed to OTC. For example, the activity of phagocytes, which are essential components of the immune system, was increased by 10-day OTC exposure in gilthead seabream (*Sparus aurata*) [29] and 3-day OTC exposure in common carp (*Cyprinus carpio*) [45]. Nevertheless, a less pronounced pattern was observed in the middle portion of the intestine compared to the previous one; however, we recorded that the highest dose of 3 μg/mL increased the transcription of all genes at 1 h post OTC exposure and this was sustained at 3, 6, 12, and 24 h. This finding is consistent with the results of Guardiola et al. (2012) [33], who also noted that the OTC concentration in the diet of gilthead seabream (*S. aurata*) or treatment doses can lead to the upregulation of immune-related genes. Likewise, late gene activation was observed in the *in vitro* experiment 24 h post exposure to OTC. In this work, 0.5 and 3 μg/mL doses evoked the most significant gene response. IκB-α and NF-κb increased their transcription levels, similar to what was noted in the previous section, with early activation at 1 h. This suggests that innate immunity in fish is a target of OTC, regardless of its mode of action. Although oxytetracycline is used to treat bacterial infections, it presents challenges in aquaculture due to its bioavailability and the side effects that it can generate at the physiological level [6,45]. It can also activate immune signaling pathways and alter internal cellular and molecular functions or mechanisms of intestinal immunity [45]. In the *in vivo* experiment, we observed a dose-dependent increase on days 3 and 5 post exposure to OTC in the anterior intestinal portion. A similar pattern to that observed in the *in vitro* experiment was discovered using this approach; both doses (high and low) activated the transcription of immune response genes, promoting the transcription of proinflammatory cytokines such as IL-6 and INF-γ and activating the NF-κb pathway. This is similar to what was observed in the midgut, where these cytokines were also activated on day 5 with both doses of OTC. This is important because mucus production is controlled by immune mediators, including leukotrienes, interferon-γ (IFN-γ), IL-9, and IL-13 [46,47], and the immune response varies along the intestinal tract. A response or pattern similar to that seen *in vitro* was observed in the hindgut. The later exposure times (6, 12, and 24 h) showed increased TLR-1 and IFN-γ gene levels, primarily with the lower dose. Overall, the gene expression results in the different tissues show interesting patterns. In particular, when the TLR/MyD88/NF-κB pathway is activated, low doses promoting the activation of biochemical innate immunity against bacterial and xenobiotic antigens such as antibiotics [32,45].

TLR-1 expression (at 1 h and on day 1) increased significantly in the foregut after exposure to OTC both *in vitro* and *in vivo*, indicating that this portion of the intestine may be susceptible to antibiotic exposure. This is consistent with the results obtained by Vargas-Chacoff et al. (2024) [32], where this increase in the TLR-1 receptor responds to early exposure to OTC and florfenicol (FLO) and promotes the transcription of MyD88 to activate NF-κb, which will then translocate to the nucleus to activate the transcription of proinflammatory cytokines IL-6 and INF-γ in the SHK-1 cell line. Shown to exhibit regional differences in pattern recognition receptors, in addition to their barrier and absorptive functions, intestinal cells express pattern recognition receptors (PRRs), which can be activated to produce mediators that recruit, activate, and condition cells of the immune system [48,49,50], as has been seen in this work. The different regions of the intestine have crucial antibacterial roles, producing antimicrobial peptides such as lysozyme and defensins and regenerating islet-derived protein IIIγ in response to interleukins or following stimulation of Toll-like receptors (TLRs) and nucleotide-binding oligomerization domain 2 (NOD2) [51,52]. This is why marked differences are seen according to response times at the genetic level by intestinal portions.

The difference in immune gene activation times observed between *in vitro* (1 h) and *in vivo* (3–5 days) assays is mainly due to physiological and contextual differences between the two models. In the *in vitro* experiment, intestinal explants were directly exposed to defined concentrations of oxytetracycline (OTC) without systemic barriers or absorption, distribution, and metabolism processes. This direct exposure allows for direct interaction of OTC with tissue epithelial and immune cells, which could explain the early activation of genes such as TLR-1, NF-κB, IL-6, and IFN-γ, observed as early as hour 1. In contrast, in the *in vivo* model, the mechanisms are more complex: when OTC is administered, it requires intestinal absorption, a systemic step, and eventually accumulation and absorption in tissues. In addition, the immune response in the whole organism involves the activation of multiple intercellular pathways, immune cell recruitment, and feedback to regulate these processes. This process requires more time, which justifies the significant activation of immune genes observed only on days 3 and 5 post exposure. This phenomenon has previously been reported in comparative immunotoxicological studies between *in vitro* and *in vivo* models in teleost fish [28,33]. It has been attributed to differences in antibiotic bioavailability, distribution kinetics, and tissue regulation.

Several studies have reported immune responses to OTC exposure in marine fish. For instance, in gilthead seabream (*S. aurata*), short-term (7 and 14 days) exposure to OTC (4 and 8 mg OTC/g feed) increased the phagocytic capacity and respiratory burst in the head kidney [33]. Our findings on antibiotics, specifically OTC, in experimental *in vitro* and *in vivo* studies, which depend on concentration and time, specifically relate to the TLR-1, MyD88, IFN-γ, and NF-κb pathways. The results are consistent with Guardiola et al. (2012) and Vargas-Chacoff et al. (2024) [32,33]. Furthermore, *in vitro* studies demonstrated that the expression of IFN-γ and IL-6 fluctuated significantly in response to different doses of OTC. In contrast, *in vivo* studies revealed that IFN-γ expression was notably higher in the hindgut, increasing significantly compared to the control. These findings suggest that cytokine regulation may vary between *in vitro* and *in vivo* conditions, underscoring the complexity of the immune response in a biologically relevant environment [31,53,54]. This indicates that the immune response is influenced by exposure duration and the dosage given. *In vivo* models offer a complex immune response, but *in vitro* assays with primary cell cultures allow us to isolate and observe direct cellular effects of antibiotics under controlled conditions. These models help detect early molecular changes and toxic thresholds. We observed responses within one hour, aligning with known pharmacokinetics. *In vitro* and *in vivo* approaches provide a fuller picture of the impact of antibiotics on fish health.

## 4. Materials and Methods

The present study used the same specimens and experimental procedures used in Muñoz et al. (2025) [55].

### 4.1. Animals

For *in vitro* experiments and injection experiments, healthy specimens of coho salmon (*O. kisutch*), weighing approximately 500.32 ± 12.4 g, were obtained from Unidad de Producción Acuícola (Universidad de Los Lagos) fish farm (Rupanco Lake, Chile).

For the *in vitro* experiments, N = 25 fish were transported to the laboratories of the Faculty of Science (Universidad Austral de Chile, Valdivia, Chile) to have portions of their intestines (foregut, midgut, and hindgut) removed and primary cells cultured.

To develop *in vivo* experiments, immature coho salmon (n = 108, 650 ± 30 g body weight [mean and SD]) were acclimated to seawater for 30 days in 9 tanks of 1000 L each, under natural conditions consisting of a 12:12 light/dark photoperiod, 12 ± 1 °C water temperature, 30 PSU, and continuously renewed and aerated water. During the acclimation period, fish were fed daily at 10:00 a.m. with commercial dry pellets for salmonids (Biomar NEW PW100_AK_CER, caliber 4.0 mm; proximate food analysis: 45–50% crude protein, 21–23% lipids, 9.5% carbohydrates, 12% ashes, 10% water, and 2.5% fiber) at 1% of their body mass.

All fish from *in vitro* and *in vivo* experiments were anesthetized with a lethal dose of 2-phenoxyethanol (1 mL/L, Sigma-Aldrich-Fluka (St. Louis, MO, USA), 77,699). The tissues were extracted from each specimen, stored, cultured, or frozen in liquid nitrogen for further analysis [11].

All experimental procedures adhered to the regulations for using laboratory animals, as outlined by the Chilean National Commission for Scientific and Technological Research (ANID) and the Universidad de Los Lagos.

### 4.2. Intestinal Primary Cell Culture Preparation

Intestinal primary cell culture preparation. For the primary culture, we obtained small pieces of tissue (approximately 10–15 mg) from the explants of the three portions (foregut, midgut, and hindgut) of *Oncorhynchus kisutch*, which were removed under aseptic conditions [56]. The intestinal portions were used for primary cell culture, which was then seeded and maintained in a six-well plate at 18 °C under an air atmosphere for at least 24 h [56,57]. The medium used for cultivating cells and tissues was Leibovitz’s L-15, with each well containing 1 mL of this medium. This medium was enhanced by adding 10% fetal bovine serum (FBS) sourced from Invitrogen (Gibco, Thermo Fisher, Waltham, MA, USA).

### 4.3. In Vitro Experimental Treatment

Twenty-four hours after seeding each of the three portions (foregut, midgut, and hindgut) of *O. kisutch*, we changed the medium to four different doses of oxytetracycline for testing (Merck; CAS No: 79–57 2) (0.25, 0.5, 1.5, and 3 μg/mL) and ensured a control group. The chosen doses are similar to those described by [28] and previously published [32,33,34,35,36,37,38,39,40,41,42,43,44,45,46,47,48,49,50,51,52,53,54,55]. For kinetic experiments at 1, 3, 6, 12, and 24 h at 18 °C, we added 1 μL/well of the antibiotic solution. Control plates contained the same medium volume without the antibiotic to avoid interference. All the experiments were run in triplicate and independently repeated twice.

### 4.4. In Vivo Experiment

All *in vivo* experiments were performed in triplicate (3 tanks per condition); the time course experiment was carried out to evaluate the effect of oxytetracycline by intraperitoneal injection using doses of 75 mg/Kg and 35 mg/Kg (concentrations used for farming), with a control group using vehicle (physiological serum) following a methodology similar to that in [28] and previously published [55]. With N = 108 fish for 5 days, samples were taken on days 1, 3, and 5 post injection. At each sampling time, 12 fish were collected (4 fish per tank, in triplicate) for each experimental condition.

### 4.5. Total RNA Extraction

Following the kinetic experiments, the cell culture medium was discarded, and 500 μL of TRIzol reagent (Sigma, St. Louis, MO, USA) was added to gather the cells. These cells were then frozen in liquid nitrogen for RNA extraction. Using TRIzol reagent, total RNA was extracted following the manufacturer’s instructions and stored at −80 °C. The concentration of the RNA was determined at a wavelength of 260 nm using a NanoDrop spectrophotometer (NanoDrop Technologies, Wilmington, DE, USA). To synthesize cDNA, 2 μg of the total RNA was used as the template for the reverse transcription process. According to standard protocols, this was performed using MMLV-RT reverse transcriptase from Promega and an oligo(dT) primer from Invitrogen.

### 4.6. RT-qPCR Analysis

The reactions were carried out using an AriaMx Real-Time PCR System (Agilent, Santa Clara, CA, USA). The cDNA (100 ng) served as the template for RT-qPCR, using Brilliant SYBR Green qPCR reagents (Stratagene, San Diego, CA, USA). Each reaction, performed in duplicate, had a total volume of 14 μL, with 6 μL of SYBR Green, 2 μL of cDNA, 1.08 μL of primer mix, and 4.92 μL of PCR water. The qPCR program used included an initial denaturation step at 95 °C for 10 min, followed by 40 cycles of denaturation at 90 °C for 10 s, annealing at 60 °C for 15 s, and extension at 72 °C for 15 s. To guarantee the specificity of the amplification process, a melting curve analysis was conducted following each PCR cycle to verify the presence of a singular PCR product. The levels of mRNA expression were determined using the comparative Ct approach (2^−ΔΔCT^), as outlined by [58,59]. The findings are expressed as the fold change in gene expression, normalized to the endogenous reference gene 18S, and compared to the control group, which consists of unstimulated cells. Table 1 lists the primers utilized for TLR-1, TLR-2, IκB-α, MyD88, NF-κB, IFN-γ, and IL-6. PCR efficiencies were assessed through linear regression analysis of the data, employing LinRegPCR software (version 11.0) [60], which plots the logarithm of serial dilutions against the threshold cycle number (ΔCT).

### 4.7. Statistical Treatment of Results

All data are presented as the mean ± standard error of the mean (SEM). Potential tank effects were tested for each physiological parameter by nesting replicate tanks within each treatment. No significant tank effects (*p* > 0.01) were witnessed in the analysis. A two-way analysis of variance (ANOVA) was used with time and treatments (doses) as factors, and the assumptions of normality, independence, and homogeneity of the residuals for the variances between groups were tested using a Shapiro–Wilk test and a Levene test. After that, a two-way ANOVA followed by Tukey’s post hoc test was used to determine whether each factor was significant and to assess the effect of treatment (control, different doses, and days) on gene expression (Table 2). All statistical analyses and graphs were performed with the software SigmaPlot 14.5.

## 5. Conclusions

**Figure 7:**
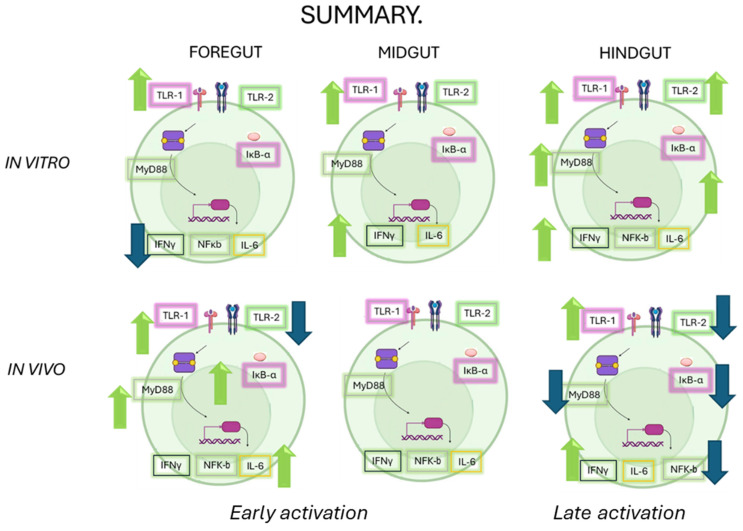
A summary of all of the responses evaluated in this work under both approaches (*in vitro* and *in vivo*). The green arrows correspond to an increase and the blue arrows correspond to a decrease in the transcripts of the immunological markers.

The role of the intestinal immune system is to maintain physiological function in the face of constant environmental challenges. There are, however, marked differences in gene expression along the length of the intestine which present distinct challenges to the immune system. As a result, although the various segments of the gastrointestinal tract can deploy a similar battery of immune functions, the exact balance between individual cell types and mechanisms varies considerably along the length of the intestine. The research presented here provides a comprehensive understanding of how OTC exposure impacts the immune response in various sections of the gut of Chilean salmon. The results highlight the complexity of the immune response, which varies not only between *in vitro* and *in vivo* conditions but also between different intestinal segments (see Figure 7). These findings have significant implications for aquaculture health and the management of antibiotic resistance, suggesting that antibiotic stewardship should be carefully considered to avoid compromising the immune response in key species for the fishing industry. Future research should comprehensively investigate the interactions between diet, antibiotics, and immune health.

## Figures and Tables

**Table 1:** Primer sequences for expression analysis.

Primer	Nucleotide Sequences (5′→3′)	Efficiency (%) Foregut	Efficiency (%) Midgut	Efficiency (%) Hindgut	GenBank No/Reference
TLR-1 Fw	TCCGGAGACGTTTCATCCCA	101.6	101.7	102.91	MF945984
TLR-1 Rv	GAGGTTCAGCGCTAACAGCA				
TLR-2 Fw	GGGTCTAACTGGGAAGCAGC	100.1	100.2	100.1	Martinez et al., 2020 [13]
TLR-2 Rv	AACGGAATGAGACGGATGGG				
MyD 88 Fw	CTTTCACAACCACCGAAGCC	102.71	100.3	101.6	Martinez et al., 2018 [17]
MyD 88 Rv	TACAAACCGAAACCGCTCCT				
INF-γ Fw	GCCGTGTGTTGGTTTTTGATTT	102.72	100.1	100.1	Martinez et al., 2018 [17]
INF-γ Rv	GTGTCTGTCTGACTGATGGTGA				
NFκ-b Fw	AAAGTGCCAGTACCAAGCCC	100.1	101.6	102.5	Martinez et al., 2018 [17]
NFκ-b Rv	CATGCTGATGAGCTACTGTTGTT				
IL-6 Fw	GAGCTACGTAACTTCCTGGTTGAC	102.1	100.3	102.1	Martinez et al., 2020 [13]
IL-6 Rv	GCAAGTTTCTACTCCAGGCCTGAT				
IkB-α Fw	TCTGCCACCAGCTGTATGA	102.5	100.1	100.1	BT074199.1
IkB-αRv	TCTGCCCGAATGTAATGTCA				
18S Fw	GTCCGGGAAACCAAAGTC	101.6	102.5	102.1	Pedro et al., 2019 [61]
18S Fw	TTGAGTCAAATTAAGCCGCA				

**Table 2:** *p*-values from the two-way ANOVA for separate time and doses and the interaction of parameters (time × doses). Statistical differences between different tissues: NS, not significant; significant, *p* < 0.01.

Tissue	Genes	*In Vitro*			*In Vivo*		
		Time	Doses	Time × Doses	Time	Doses	Time × Doses
Foregut	TLR-1	<0.0001	<0.0001	<0.0001	<0.0001	<0.0001	<0.0001
Midgut	TLR-1	<0.0001	<0.0001	<0.0001	<0.0001	<0.0001	<0.0001
Hindgut	TLR-1	<0.0001	<0.0001	<0.0001	<0.0001	NS	NS
							
Foregut	TLR-2	<0.0001	NS	<0.0001	<0.0001	<0.0001	<0.0001
Midgut	TLR-2	<0.0001	<0.0001	<0.0001	NS	0.0077	<0.0001
Hindgut	TLR-2	<0.0001	<0.0001	<0.0001	<0.0001	<0.0001	<0.0001
							
Foregut	MyD 88	<0.0001	0.0001	<0.0001	<0.0001	<0.0001	<0.0001
Midgut	MyD 88	<0.0001	<0.0001	<0.0001	NS	<0.0001	0.0004
Hindgut	MyD 88	<0.0001	<0.0001	<0.0001	0.0002	NS	0.0156
							
Foregut	IkB-α	<0.0001	<0.0001	<0.0001	<0.0001	<0.0001	0.0006
Midgut	IkB-α	0.0037	0.0111	<0.0001	0.0008	<0.0001	0.0001
Hindgut	IkB-α	<0.0001	<0.0001	<0.0001	<0.0001	0.0048	<0.0001
							
Foregut	INF-γ	<0.0001	<0.0001	<0.0001	<0.0001	0.0105	<0.0001
Midgut	INF-γ	<0.0001	<0.0001	<0.0001	<0.0001	<0.0001	<0.0001
Hindgut	INF-γ	<0.0001	<0.0001	<0.0001	<0.0001	<0.0001	<0.0001
							
Foregut	IL-6	<0.0001	<0.0001	<0.0001	<0.0001	<0.0001	0.0019
Midgut	IL-6	<0.0001	<0.0001	<0.0001	<0.0001	0.0103	<0.0001
Hindgut	IL-6	<0.0001	<0.0001	<0.0001	<0.0001	NS	0.0002
							
Foregut	NFκ-b	<0.0001	<0.0001	<0.0001	<0.0001	NS	<0.0001
Midgut	NFκ-b	<0.0001	<0.0001	<0.0001	0.0063	NS	NS
Hindgut	NFκ-b	<0.0001	<0.0001	<0.0001	NS	<0.0001	NS

## Data Availability

Data availability statements are available on request.
